# Ecological Risks Arising from the Impact of Large-scale Afforestation on the Regional Water Supply Balance in Southwest China

**DOI:** 10.1038/s41598-020-61108-w

**Published:** 2020-03-05

**Authors:** Yang Xiao, Qiang Xiao, Xuefeng Sun

**Affiliations:** 1 College of Biology and Environmental Sciences, Jishou University, Jishou, 416000 China; 2 Chongqing University of Arts and Sciences, Chongqing, 402160 China

**Keywords:** Phenology, Ecosystem services, Phenology

## Abstract

Ecological degradation impedes sustainable development in Southwest China, and artificial afforestation has been a key strategy of the Government of China to effectively curb it. However, the water consumed by large-scale afforestation has a huge impact on water supply in Southwest China, which also arise a new challenge causing severe drought here recently. In order to determine the impact of artificial afforestation on the region, this study conducts spatiotemporal, abrupt change, and correlation and regression analyses. Results show that although water resources fluctuate, they exhibit a general declining trend. Especially after 2000, water resources showed a significant downward trend. In terms of spatial tendency, there has been an obvious decrease in water resources in the Sichuan Basin, the mountainous region of eastern Chongqing, and the Yunnan–Guizhou Plateau. Due to extensive afforestation and vegetation cover growth, there has been an increase in most trends of forest-shrub ecosystems, resulting in substantially enhanced evapotranspiration effects. The uptake of water by afforestation in Southwest China could reach 40.42 billion m^3^, which is equivalent to 10.69% of its annul available water supply (1980–2015). Generally, afforestation disrupts the water balance of the region. This study recommends substituting afforestation for natural restoration or, at the very least, selecting vegetation that requires less water for the restoration of the ecological environment of Southwest China, which provide scientific method for regional sustainable development.

## Introduction

The IPCC Fifth Assessment Report on climate change stated that global surface temperatures rose by 0.85 °C from 1880 to 2012 (from 0.65 °C to 1.06 °C, respectively). Global warming leads to frequent extreme weather and climate events and impacts both the water supply and the ecological environments of numerous countries^1,2^. Severe droughts are caused by a decrease in precipitation, which leads to shortages in water supplies, and such droughts are harmful to life, production, and ecology. Moreover, events such as these have dual attributes, that is, natural attributes and social attributes. This is an important factor which restricts the sustainable development of societies^3^. Since the twenty-first century, drought events in Southwest China have moved into a period of “high incidence”. With increases in interannual and annual variation in rainfall in Southwest China, the frequency of droughts have increased, and years of serious and catastrophic drought events in different regions and during different seasons have subsequently increased^4^. Thus, strengthening research on drought characteristics and causes in Southwest China as well as the level of response, providing a scientific basis for decisions on disaster prevention and mitigation, have important practical implications^5,6^.

Since 2010, there have been many serious drought events reported in Southwest China, including Yunnan, Guizhou, and Sichuan provinces and the municipality of Chongqing, which have attracted wide attention from all walks of life^7^. The drought from 2005 to 2016 affected 6 368 700 hm^2^ of farmland throughout the whole of Southwest China and 5 010 700 hm^2^ of crops^8^. This effected access to available drinking water for 20 199 000 people. Direct agricultural losses in the hardest-hit areas of Yunnan Province exceeded 30 billion yuan^9^.

There are many opinions concerning the causes of drought in Southwest China. The lagging development theory of water conservancy believes that the aging or the lack of water conservancy facilities result in insufficient drought resistance; thus, such areas are vulnerable to drought^10^. The climate change theory states that the El Niño phenomenon causes insufficient water distribution in Southwest China, and, coupled with high local temperatures and high evaporation rates, drought will result. The reasoning behind the irrational utilization theory of water resources is that local economic development exceeds the carrying capacity of local water resources, resulting in low utilization efficiency of resources and high water pollution^11^. Qualitative analysis has been the focus of many discussions on this issue, and quantitative evaluation has focused on causes related to climate^12^.

Southwest China is rich in biodiversity and wide range of ecosystem services. It is a strong support for the sustainable development of the Yangtze River Basin, the Pearl River Basin and Mekong River Basin. The area as an eco-fragile region is also known for its severe soil erosion and the tendency to develop rocky desertification. This is due to carbonate rocks being exposed to the environment in combination with high solubility and low physical weathering rate. The formation of soil is slow^13^. In order to protect the fragile ecosystem, reduce natural disasters and curb ecosystem degradation, the Chinese central government worked with local governmenthave recently launched a series of ecological policies in Southwest China, such as the Slope Land Conversion Project (SLCP), China’s Natural Forest Protection Project (NFPP), and the River Shelterbelt Project,. At the same time, the ‘Go West’ policy has promoted large-scale and rapid urban development. The regional land cover has changed significantly^13^.

Due to the combination of man-made and natural factors, the Plateau has suffered serious ecological degradation. To alleviate this phenomenon and protect regional water resources, the Chinese government has implemented large-scale afforestation in Southwest China. For example, from 1949 to 2011, the area of artificial forest increased by more than 16100 square kilometers, of which the construction area of protective forest and water conservancy forest accounted for 87.0% and 9.3% of the afforestation area, respectively. Although the vegetation coverage has improved significant in recent years, few people have focused the potential impact of ecological policies scheme on the downstream water supply^14^. There remains considerable controversy in academic circles concerning the effects of artificial and natural forests in response to drought. Artificial and natural forests are both widely distributed in Southwest China; accordingly, this region is the ideal place to study differences in natural and artificial forests in response to drought.

In our research, we demonstrate the potential impact of lagre-scale afforestation on the water use efficiency of residents living in Southwest China, and the cause of this phenomenon. In order to support our analysis, we calculated the impacts of afforestation and restoration based on natural vegetation protection strategies on regional water balance. In summary, the establishment of appropriate ecological management practices for water supply conservation is an important and urgent scientific step for promoting the effective use of water resources and reducing the pressure of water scarcity in the ecological environment.

## Results

### The increasing water scarcity in Southwest China

**Figure 1:**
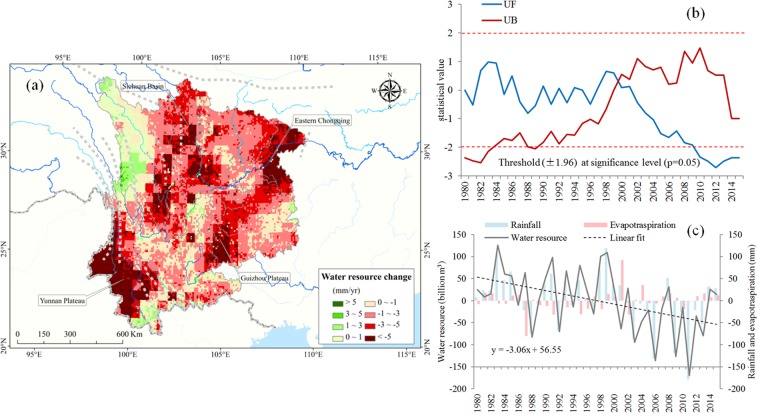
Study on the spatial-temporal pattern, change trend and mutation of water resources in Southwest China. (**a**) The change characteristics of water resources from 1980 to 2015, and the regions indicated by cross marks show obvious changes (P < 0.05). (**b**) The turning point of trend determined by Mann Kendall test. (**c**) Annual trends in water resources, rainfall and evapotranspiration anomalies (distance mean value) from 1980 to 2015. The “Red” represents the area of decreasing water resource, and “Green” represents the area of increasing water resource in (**a**); The “UF” represents the positive sequence, and “UB” represents the inverse sequence in (**b**), and the intersection between reliability lines indicates the mutation time in (**b**); The dashed line in (**c**) represents the linear trend of distance mean value of water resource, with slope −3.06 (billion m^3^ yr^−1^). We created this figure in using ArcGIS 10.3 for maps (URL: http://www.esri.com/). Water resources data is calculated based on water balance theory in the methods section.

From 1980 to 2015, although water resources fluctuated, the overall trend was downward (with a decrease of 3.06 billion m^3^/yr). We found that there was a close relationship between water resources and precipitation anomalies. In addition, during the whole study period, the water resource anomaly has a significant downward trend (Fig. 1a**)**. On a regional scale, almost all important domains (Sichuan, Yunnan, and Guizhou provinces and the municipality of Chongqing) have undergone severe water shortages between 1980 and 2015 (Fig. 1b), which is consistent with China’s drought report in recent years^15^. In total, water resources reduced significantly in the Sichuan Basin, the mountainous region of eastern Chongqing and the Yunnan–Guizhou Plateau. In contrast, the total amount of water resources in Hengduan Mountains has increased. To assess the water supply change in time series, we use Mann Kendall trend test to calculate the turning point of the trend. As shown in Fig. 1c, we have identified two distinct trends, 1980–2000 and 2000–2015.water resources fluctuate significantly(slope = 0.69 billion m^3^/yr) prior to the year 2000. Subsequently, water resources again gradually declined (slope = −3.64 billion m^3^/yr).

In addition, The characteristics of water resources in different time series in Southwest China were shown in Table 1. The annual average water storage in Yunnan, Sichuan, and Guizhou provinces and the municipality of Chongqing are 311.4 mm, 309.98 mm, 409.5 mm, and 482.22 mm, respectively; and mean coefficients of variation are 0.32, 0.21, 0.32, and 0.31, respectively, from 1980 to 2015.

**Table 1:** Characteristics of water supply during different decades in Southwest China.

Water storage	Yunnan		Sichuan		Guizhou		Chongqing		Total area	
Series	Average (mm)	CV	Average (mm)	CV	Average (mm)	CV	Average (mm)	CV	Average (mm)	CV
1980s	316.11	0.31	348.75	0.15	404.51	0.31	556.01	0.31	362.1	0.14
1990s	377.66	0.22	315.96	0.25	489.30	0.14	485.38	0.27	375.73	0.15
2000s	289.87	0.33	268.22	0.20	361.37	0.35	422.96	0.34	301.32	0.18
2010s	228.99	0.32	305.01	0.18	365.06	0.48	452.72	0.25	300.21	0.21
1980–2015	311.40	0.32	309.98	0.21	409.50	0.32	482.22	0.31	338.69	0.19

#### Causes for decreases in water supply in Southwest China

**Figure 2:**
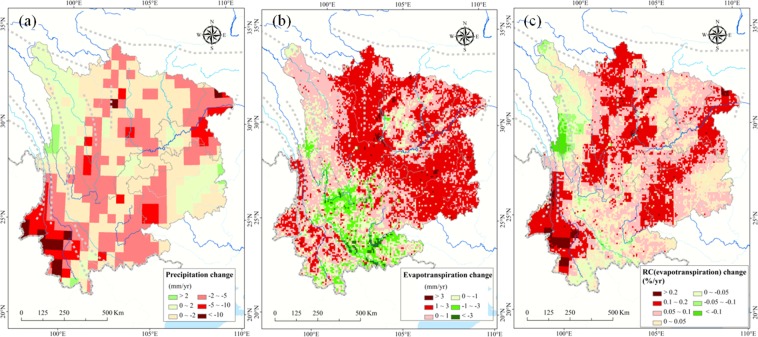
Change patterns of precipitation (**a**) and evapotranspiration (**b**) and the contribution of evapotranspiration (**c**) in Southwest China. RC denotes the relative contributions of factors (precipitation and evapotranspiration). The “Red” represents the area of decreasing precipitation, and “Green” represents the area of increasing precipitation in (**a**). To the contrary, the “Red” represents the area of increasing evapotranspiration or its contribution, and “Green” represents their decreasing trend in (**b**,**c**). We created this figure in using ArcGIS 10.3 for maps (URL: http://www.esri.com/). The trend of factors is calculated based on a linear regression model in the methods section. RC is calculated based on the contribution equation in methods section.

Figure 2 shows that declining precipitation or rising evapotranspiration or combination of both result in the growing water scarcity observed throughout most of Southwest China (greater than 85% of the domain). The largest decline in precipitation (greater than 5 mm/yr) was in the Yunnan–Guizhou Plateau, and the greatest increases in evapotranspiration were mainly distributed throughout sections of the northeastern and southwestern plateau (Fig. 2a,b), where water resources have dramatically decreased (greater than 5 mm/yr) since 1980. The temporal pattern of the contribution of evapotranspiration to water scarcity is shown in Fig. 2c. Due to extensive afforestation and the subsequent growth in vegetation cover, most forest-shrub ecosystems (approximately 82%) exhibited increasing trends in NDVI, which resulted in substantially enhanced evapotranspiration effects.

We estimated the growing water scarcity resulting from interactions between climate change and anthropogenic activities using contribution equations^16^, and our results are provided in Table 2. From 1980 to 2015, the evapotranspiration effect reduced water resources throughout almost the entire domain, especially in the municipality of Chongqing and Guizhou Province, and changes in evapotranspiration resulted in the greatest contributions to water scarcity (0.09%/yr and 0.08%/yr, respectively). Evapotranspiration factors that controlled changes in water resources varied for the different subperiods. Evapotranspiration contributions in Yunnan and Guizhou provinces between 1980 and 2001 showed decreasing trends; however, from 2002 to 2015, these contributing trends abruptly changed (0.19%/yr and 0.01%/yr, respectively). Table 2 shows that the contribution rate of evapotranspiration to water resources revealed an increasing trend and, conversely, the contribution rate of precipitation revealed a decreasing trend, which indicated that evapotranspiration was gradually replacing precipitation as it pertains to the contribution rate to local water supply, and that evapotranspiration was the main cause for local droughts.

**Table 2:** Contributions of evapotranspiration to the reduction of water supply in Southwest China.

Series	Yunnan		Sichuan		Guizhou		Chongqing		Total area	
	Average (%)	Change (%/yr)	Average (%)	Change (%/yr)	Average (%)	Change (%/yr)	Average (%)	Change (%/yr)	Average (%)	Change (%/yr)
1980–2001	41.49	−0.13	39.93	0.11	38.13	−0.04	36.75	0.2	39.93	0.01
2002–2015	43.59	0.19	41.02	−0.13	40.28	0.01	37.91	0.03	41.52	0.01
1980–2015	42.31	0.06	40.35	0.06	38.97	0.08	37.2	0.09	40.55	0.07

**Figure 3:**
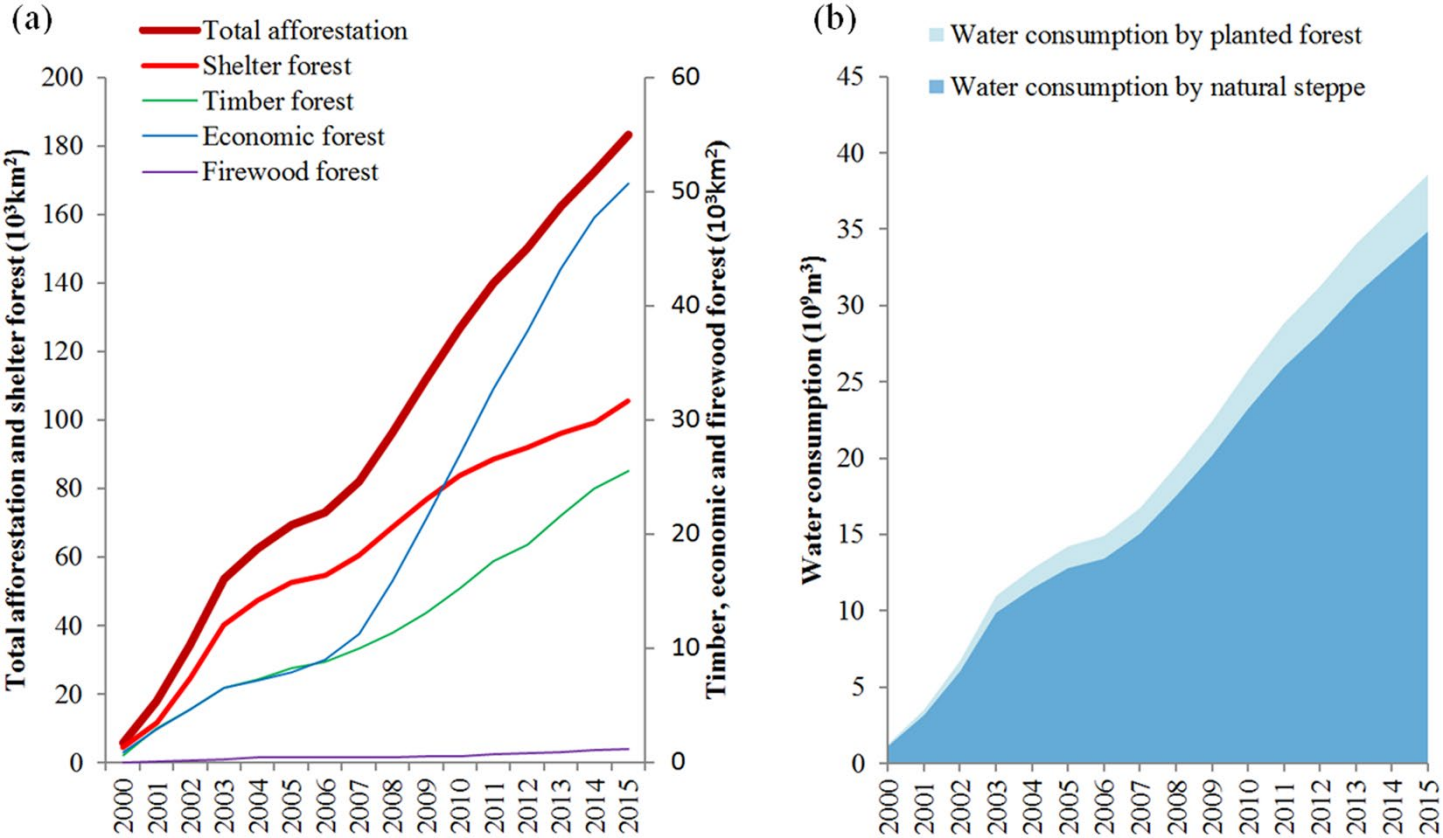
The afforested area in Southwest China from 2000 to 2015 (**a**) and Water consumption estimation for planting artificial vegetation and planting natural vegetation (**b**). Shelter forest denotes protecting ecological systems, including soil and water conservation forest, windbreak forest and sand fixation forest, etc.; Timber forest denotes wood production forest, including bamboo forests production; Economic forest denotes the trees mainly used for the production of fruits, industrial raw materials and medicinal materials etc.; Firewood forest denotes the forest used for the production of fuel.

In order to cope with the serious ecological degradation and improving well-being of local residents since 2000, the Chinese government has implemented a series of ecological policies to improve the ecosystem services, such as the NFPP and SLCP programs. Afforestation means to increase vegetation coverage by expanding forest-shrub area artificially.The planted area of shelterbelts account for 57.55% of total afforestation area in Southwest China (Fig. 3a) and almost half of shelterbelts distributed in Yunnan and Sichuan provinces (Table 3), which means that under this strategy runoff regulations and soil retention have become the primary goal of the government. The planting of non-native species are generally encouraged in regions where the SLCP and NFPP programmes predominate, although the actual survival rate is less than 20% calculating based on the China forest resources report (http://www.forestry.gov.cn/). In this study, we find that the water consumption of plantation since 2000 in Southwest China is 40.42 billion m^3^, which is equivalent to 10.69% (Fig. 3b) of water supply in the whole year. By contrast, we find that under the practice of planting natural vegetation (i.e., the planting of dominant native vegetation in most afforested areas), a large quantity of water would still be consumed, reaching 35.31 billion m^3^ by 2015, which is account fort 9.33% of the annul water resources.

**Table 3:** Area ratio of planted forests-shrub in the different provinces and municipality in Southwest China. Unit: %.

Province	Planted forests	Timber forest	Economic forest	Shelter forest	Firewood forest
Chongqing	13.91	18.24	5.79	14.87	18.72
Sichuan	30.38	28.57	10.39	34.22	27.88
Guizhou	19.69	18.15	12.43	20.63	18.32
Yunnan	36.01	35.04	71.40	30.29	35.08

A large number of plantations in Southwest China will consume a lot of water resources. Even planting natural forests, such those proposed by ecological engineering policies, will still consume a large amount of water resources; however, it would be about 5 billion m^3^ less water compared to artificial forests. Therefore, the implementation of scientifically-sound and appropriate management practices of ecological engineering would be an important practical measure in maintaining regional ecological water supply security.

## Discussion

In order to control rocky desertification and maintain ecological environment, the State Forestry Administration has planted 640000 hectares of trees in Southwest China since 1952. However, planting large numbers of artificial forests absorb large amounts of available water from the soil, leading to droughts and insufficient water on the surface of the soil, which is the most common phenomenon seen in the afforested area of Southwest China, resulting in a significant impact on the water supply in the basin^17^. Compared to natural forest systems, artificial afforestation leads to an increase in evapotranspiration. Recent studies have discovered a new impact caused by afforestation, that is, that artificial forests have a superimposed effect on drought and climate change in the research area. Our study confirms this new characteristic of artificial afforestation. Furthermore, decreases in precipitation or increases in evapotranspiration have caused water shortages in most parts of Southwest China^18,19^. By comparing evapotranspiration between artificial and natural forests, we concluded that the evapotranspiration rate of artificial forests is higher compared to natural forests. We found the most significant decreases in precipitation in the Yunnan–Guizhou Plateau (greater than 5 mm/yr), while we determined that the maximum evaporation rate was distributed in certain areas of the northeastern and southwestern plateau^20^. These findings coincide with our research hypothesis. This phenomenon was the result of extensive afforestation and vegetation growth, where most forest and shrub ecosystems exhibited increasing NDVI, which in itself will greatly increase overall evapotranspiration^21^.

Due to the interactions between climate change and anthropogenic activities, water supply is in short provision in the study region. From 1980 to 2015, evapotranspiration decreased throughout almost the entire region, and variation in evapotranspiration had the greatest contribution to water scarcity (0.09%/yr and 0.08%/yr, respectively). During the different sub periods, the amount of evapotranspiration controlling water resources changes varied^22^. From 1980 to 2000, for example, evapotranspiration contributions in Yunnan and Guizhou provinces exhibited a downward trend, but their contributions rapidly changed from 2000 to 2015. The contribution rate of evapotranspiration to available water resources exhibited an increasing trend, and, conversely, the contribution of precipitation exhibited a decreasing trend, which indicated that evapotranspiration was gradually replacing precipitation with regards to its total contribution to local water supply^23^. As stated earlier, this study found that evapotranspiration had a superimposed effect on drought in Southwest China. In addition, the contribution of evapotranspiration and precipitation to water supply changed over the past 30 yr^24^. These results confirmed our hypothesis that afforestation has a superimposed effect on drought in Southwest China.

In order to improve human welfare, China implemented a series of national afforestation policies, including the NFPP and SLCP. The government’s original intention was to increase vegetation and help local communities to rise above poverty levels. When evapotranspiration increases, however, vegetation is forced to subsist on groundwater extraction, which causes the depletion of near surface water and the decrease of groundwater level, thus resulting the water shortage of local vegetation^25^. In addition, the increasing population and the rapidly developing economy in the South Western area of the study region have increased overall water consumption levels, resulting in the further depletion of water resources and the expansion of water resources gaps^26^. This situation may lead to risks in competition for water resources in the future, such as between Yunnan and Guizhou provinces and between Sichuan Province and the municipality of Chongqing^27^. As stated, afforestation measures were implemented to improve vegetation coverage and increase forest area. Afforested area in Southwest China accounts for 57.55% of the total afforested area of the region, which means that runoff regulations and soil and water conservation have both become the primary goal of the government. Although the purpose of afforestation was the government’s way to protect the environment, in actual fact it has interfered with the ecological environment in Southwest China.

Recent studies have shown that drought in Southwest China is the result of a combination of rainfall reduction and large-scale afforestation^28^. Although the increased evapotranspiration associated with afforestation can improve the total relative humidity of local atmosphere, which could increases the rainfall in the low wind direction of plantation. It remains to be confirmed whether this will happen^29^. The principle of caution tells us that the concept of large-scale afforestation should be strictly reviewed. Before the implementation of reforestation, we should protect and reconstruct the natural ecosystem of Southwest China. In addition, decisions of ecological engineering should be to select tree species and grass species to lower water consumption (evapotranspiration) to reduce overall water consumption^30^. This type of water conservation will reduce many ecological risks, including the risk of the unreasonable allocation of limited water resources in a number of economic and social fields^31^. To reduce such risks, the scientific understanding of water supply in Southwest China must be improved.

The environmental goals set by the government cannot be separated from the support provided by certain types of economic development^32^. Without such support, local residents would bypass environmental restrictions on their original land, food sources and maintenance obligations. This will in turn result in a ‘poverty trap’. Environmental improvements and the appropriate governance of poverty are complementary^33^. The key to making investments in environment assets and management is to achieve goals of environmental cost-effectiveness and poverty alleviation. In this study, we found that 38.59 billion cubic meters of water were consumed by plantations in Southwest China, equivalent to 10.21% of the total water resources between 1980 and 2015. In addition, the yearly decrease observed in water resources and the yearly increase observed in evapotranspiration have alarmed those that implemented and control large-scale afforestation projects in Southwest China. Therefore, the key in reversing this problem is to improve ecological benefits without harming the ecological environment^34^.

## Conclusions

Our results showed that afforestation in Southwest China has a certain effect on the regional water supply balance, and that increases in the vegetation index throughout the last 30 yr exacerbated the impact of drought in Southwest China. Southwest China has abundant and adequate rainfall. However, the plantation in Yunnan, Guizhou, Sichuan and other places also led to the increase of total evapotranspiration, which exceeded the past evapotranspiration and led to the consumption of local water resources. These ecological risks are mainly originated from afforestation and land use changes, which focus on the short-term economic return but lose sight of the consequences of long-term ecological impact. We found that the key to resolving the water supply challenge in Southwest China is to improve the utilization efficiency of available water, which is one of the most effective management strategies to protect and maintain the current ecological environment and ecosystem.

Restrictions on human activities in Southwest China would be greatly significant for the protection of water supply in Southwest China. Changes in how we use water resources are critical to maintaining them, which is in itself a strong indicator of the problems of water supply security in Southwest China. The ecological risks arising from the impact of large-scale afforestation on the regional water supply balance in this study sheds some light on the fact that afforestation should take into account regional water cycles and climate change. At the same time, how to improve the efficiency of water use to strengthen the vegetation restoration in Southwest China while avoiding the potential risk is put forward.

## Materials and Methods

### Study area

**Figure 4:**
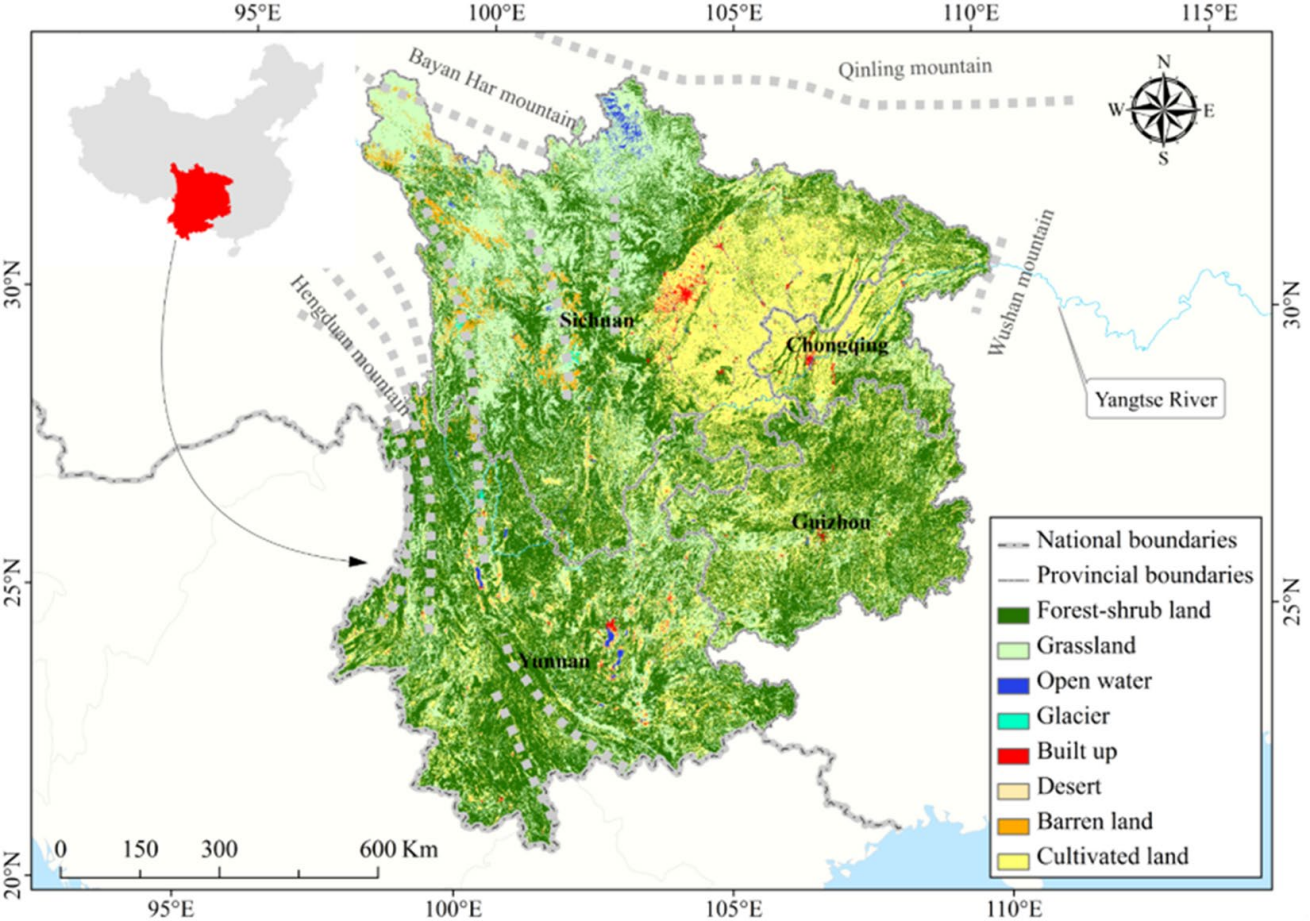
Location and ecosystem type distribution map for Southwest China. We created this figure in using ArcGIS 10.3 for maps (URL: http://www.esri.com/). The “Gray” dotted line represents the mountains marked name in nearby label. Different colors represent different ecosystem types, such as “Dark Green” represents the forest ecosystem.

Southwest China (21°08′–33°41′N, 97°31′–110°11′E), consisting of Sichuan Basin, Yunnan Guizhou Plateau and Southeast of Qinghai Tibet Plateau, mainly located in the southwestern hinterland of China. It covers three provinces and one major municipality, that is, Sichuan, Guizhou, and Yunnan provinces and Chongqing Municipality, comprising of a total of 436 counties. The total area could reach 1.13 million km^2^, accounting for 11.75% of the national terrestrial area. The main geomorphic forms are plateau, mountain, hill and basin, and the hilly area accounts for more than 80% of the study area. And, karst landforms (e.g. trough valley, peak cluster depression and rift basin) are widely distributed in this area. For most areas, Southwest China experiences humid subtropical monsoon climate with an annual mean temperature of about 20 °C. Moreover, the mean annual precipitation is greater than 1000 mm, which mainly occurs in the form of frequent rainstorms. The main ecosystem types in Southwest China are forest shrub, grassland and farmland. (Fig. 4).

### Data sources

We obtained meteorological data (monthly precipitation, monthly temperature, sunshine duration, and etc.) during the time period 1980–2015 from the Chinese National Meteorological Information Center (http://data.cma.cn). We obtained ecosystem type data with an average overall accuracy greater than 94%^35^ in China (1980, 1990, 1995, 2000, 2005, 2010, 2015) from the National Earth System Science Data Sharing Infrastructure, Chinese Academy of Science (http://www.geodata.cn). Digital elevation model (DEM) data originated from the Shuttle Radar Topography Mission was collected from the NASA Earth Science Data (https://earthdata.nasa.gov/). The Normalized Difference Vegetation Index (NDVI) dataset originated fromGlobal Inventory Modeling and Mapping Studies (GIMMS) covering the period from 1982 to 2015 was obtained from the Ecological Forecasting Lab of NASA’s Ames Research Center (https://ecocast.arc.nasa.gov). The other socio-economic and hydrological data, such as population, Gross Domestic Production (GDP), afforestation, water resource, etc., were collected from China Statistical Yearbook (http://tongji.cnki.net), China Forestry Statistical Yearbook (http://tongji.cnki.net/), and China’s water resource bulletin (1998–2015, http://www.mwr.gov.cn/).

### Water supply quantification

In order to quantify water supply, we apply the water balance equation to the hydro series (1980–2015),which can be described as follows^36^

1 $$Q=P-E{T}_{a}-\varDelta S$$

where *Q* indicates the amount of water supply namely water resource (mm); *P* refers to precipitation (mm), and *ET*_*a*_ refers to actual evapotranspiration (mm); and *ΔS* indicates to basin water supply change (mm), which is generally assumed to be zero in a long term.

### Evapotranspiration factor (ET)

Based on the assumption similar to Budyko^36^, the actual evapotranspiration can be calculated as follows:

2 $$ET=\frac{P\times E{T}_{p}}{{({P}^{n}+E{T}_{p}^{n})}^{1/n}}$$

Where ET indicates the actual evapotranspiration (mm), and the control parameters of n model represent the landscape characteristics of the watershed, which is mainly related to the soil, terrain and vegetation attributes^37^. ET_P_ as the potential evapotranspiration (mm), can be calculated using the Priestley Taylor (PT) equation^38^:

3 $$E{T}_{p}=\alpha \times \frac{\varDelta }{\varDelta +\gamma }\times ({R}_{n}-G)$$

4 $$\varDelta =\frac{4098\times (0.6108\exp (\frac{17.27T}{T+237.3}))}{{(T+237.3)}^{2}}$$

5 $$\gamma =0.665\times {10}^{-3}\times 101.3\times {(\frac{293-0.0065H}{293})}^{5.26}$$

where *α* refers to the PT coefficient (1.26) for open water and saturated land^38^; *Δ* is *t*he slope of the saturation vapor pressure curve (kPa; °C^−1^); and *γ* refers to the psychrometric constant (kPa; °C^−1^). *R*_*n*_ is the net absorbed radiation at the surface in MJ m^−2^, *G* indicates the downward soil heat flux in MJ m^−2^ (*G* = 0.26*Rn*), *T* is mean air temperature (°C). *H* refers elevation above sea level (m).

### Contributions quantification

The quantificational identification of contribution of each factor to the reduction of water supply can be performed using the equation presenting as follows^16^:

$$T(Q)=C(P)+C(ET)$$

where *T(Q)* refers to actual water supply (Q) of linear trend, *C(P)* and *C(ET)* denote the contributions of changes in Precipitation (P) and Evapotranspiration (ET), respectively to the variation in water supply.

The relative contribution of factor P and ET can be estimated as follows:

$$RC\_({x}_{i})=\frac{C\_({x}_{i})}{T\_(Q)}\times 100 \% $$

where xi represents each factor of the above two variables (P and ET)

### Trends analysis

To detect the trend of water supply variation during the research period, the least square linear regression model can be used^39^. Fit the linear equation of water supply variables with time (year), and get the trend of each pixel change.

### Abrupt change analysis

We mainly use Mann Kendall method to analyze the mutation of water supply^40^. For the Mann Kendall test of mutation, under the premise of positive sequence curve (UF_k_), cross the critical ratio reliability line. If there is only an obvious intersection between the positive sequence (UF) and the reverse sequence (UB generated by the reverse data sequence of UF) in the reliability line, it is the mutation point, which is statistically significant. On the other hand, for example, if If the intersection is outside the reliability line, or there are many obvious intersections between lines, the mutation point cannot be determined. In the latter case, we apply the Mann Kendall method to different length sequences based on the mobile t-test technology. For different sequences, if there is still a mutation point, we can confirm that this point is the confirmed mutation point.

### Model validation

**Figure 5:**
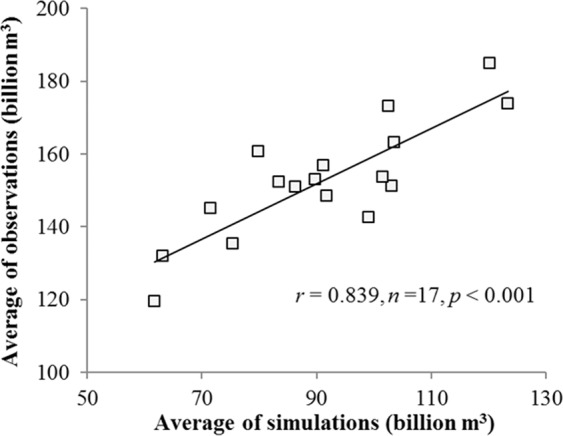
Water supply verification results from 1998 to 2015. Water resources observation data was collected from China Water Resources Bulletin (1998–2015, http://www.mwr.gov.cn/).

In order to verify the reliability of simulated water supply, the observation data (1998–2015) obtained from China water resources bulletin was uesd to verify the simulation results. The simulation results of water supply are in good agreement with the observed values (r = 0.84; P < 0.001; Fig. 5).
